# Changes in expression of transforming growth factor beta mRNA isoforms in patients undergoing tamoxifen therapy - reply

**Published:** 1997

**Authors:** J MacCallum, WR Miller


MacCallum J, Keen JC, Bartlett JMS, Thompson AM, Dixon JM and Miller WR (1996) Changes in expression of transforming growth factor beta mRNA isoforms in patients undergoing tamoxifen therapy. *Br J Cancer* **74**: 474–478

Perry RR, Kang Y and Greaves BR (1995) Relationship between tamoxifen-induced transforming growth factor β1 expression, cytostasis and apoptosis in human breast cancer cells. *Br J Cancer* **72**: 1441–1446

### Changes in expression of transforming growth factor beta mRNA isoforms in patients undergoing tamoxifen therapy

**Reply to the letter from Benson and Colletta**

### Sir

The controversy surrounding the complex role of transforming growth factor (TGF-β) in breast cancer is once again highlighted in the letter of Mr JR Benson and Dr AA Colletta. The comments made are informative and, while we would concur with many of them, we feel that some additional response is appropriate to clarify some of the issues raised.

First of all, it is important to emphasize that the aim of our study was to determine the effect of tamoxifen on TGF-β expression in breast cancers from patients whose tumours could be accurately assessed for response to therapy. It was not a study to elucidate the role of TGF-β in carcinogenesis or to assess expression in different stages of disease, although we have already published data on the latter (MacCallum et al, 1994). Although the study is limited to a defined group of patients, this is an important cohort, and one in which we could obtain sequential samples of tumour and accurately assess response of these same lesions to tamoxifen treatment (Incidentally, the rationale for using ultrasound as an accurate assessment of tumour size has already been published by Forouhi et al, 1994).

Secondly, we believe we have correctly shown conservatism in terms of attributing differences between sequential samples of the same tumour to the effects of tamoxifen. It is essential that inherent variations of methodology and tumour heterogeneity are assessed and realistically taken into account. Having done this in the present study, it was reassuring that, with regard to TGF-β2 not only was it more likely for expression to be higher in tamoxifen-treated biopsies but also that this pattern was exclusive to responding tumours. We have therefore been satisfied that these effects were mediated by tamoxifen. However, the direction of effects of treatment on TGF-β1 were almost equally increases or decreases, and there was no statistical difference in patterns between responders and non-responders. We have therefore been reluctant to claim dogmatically that these influences are caused by tamoxifen, despite a degree of change exceeding that of our controls.

Nevertheless, we have discussed the possibility that tamoxifen might more commonly induce the expression of TGF-β1 in breast cancers, an effect which may not have been apparent in our study for a variety of reasons (MacCallum et al, 1996). The exhaustion of stromal induction of TGF-β, as suggested by JR Benson and AA Colletta, is also possible. However, that the stroma is the primary source of TGF-β is controversial; both we and others have shown that TGF-β appears to be synthesized predominantly within epithelial cells of breast cancers (Auvinen et al, 1995; MacCallum et al, 1995; Walker and Gallagher, 1995). This is not necessarily at odds with the apparent increased staining of TGF-β1 in stroma following primary tamoxifen therapy, as reported by Butta et al (1992), if the growth factor was synthesized and secreted by epithelial cells, but sequestered by the stromal compartment. Indeed, if tamoxifen causes the death of epithelial cells, there might be an impression of upregulation in residual stroma. We would agree however, that simultaneous measurements of TGF-β protein and mRNA would give an additional dimension to these studies. Our immunohistochemical investigations are currently under way, and preliminary data suggest that both TGF-β1 and TGF-β2 predominantly localize to the epithelium.

In vitro studies using cell lines and animal models have yielded important understanding of the role of TGF-β in breast cancer, but ultimately it is necessary to look at appropriate clinical material. Such translational studies can be difficult to perform and may produce results that are subject to variable interpretation depending on perspective. However, in carrying out the reported study, we believe that we have not only generated meaningful results, but have been objective in deriving our conclusions.

*J MacCallum and WR Miller*
*ICRF Medical Oncology Unit, Western General Hospital*
*Edinburgh EH4 2XU, UK*

### REFERENCES

Auvinen P, Lipponen P, Johanssom R and Syrjanen K (1995) Prognostic significance of TGF-β2 expression in female breast cancer. *Eur J Cancer* **31A**: 851

© Cancer Research Campaign 1997

Butta A, Maclennan K, Flanders KC, Sacks NPM, Smith I, McKinna A, Dowsett M, Wakefield LM, Sporn MB, Baum M and Colletta AA (1992) Induction of transforming growth factor β1 in human breast cancer *in vivo* following tamoxifen treatment. *Cancer Res* **52**: 4261–4264

MacCallum J, Bartlett JMS, Thompson AM, Keen JC, Dixon JM and Miller WR (1994) Expression of TGFβ mRNA isoforms in breast cancer. *Br J Cancer* **69**, 1006–1009

MacCallum J, Poulsom R, Hanby AM and Miller WR (1995) Expression and distribution of TGFβ mRNA isoforms in a small group of human breast cancers examined by *in situ* hybridization. *The Breast* **4** 289–296

MacCallum J, Keen JC, Bartlett JMS, Thompson AM, Dixon JM and Miller WR (1996) Changes in expression of transforming growth factor beta mRNA isoforms in patients undergoing tamoxifen therapy. *Br J Cancer* **74**: 474–478

Walker RA and Gallagher B (1995) Determination of transforming growth factor $beta_1$ mRNA expression in breast carcinomas by *in situ* hybridisation. *J Pathol* **177**: 123–127


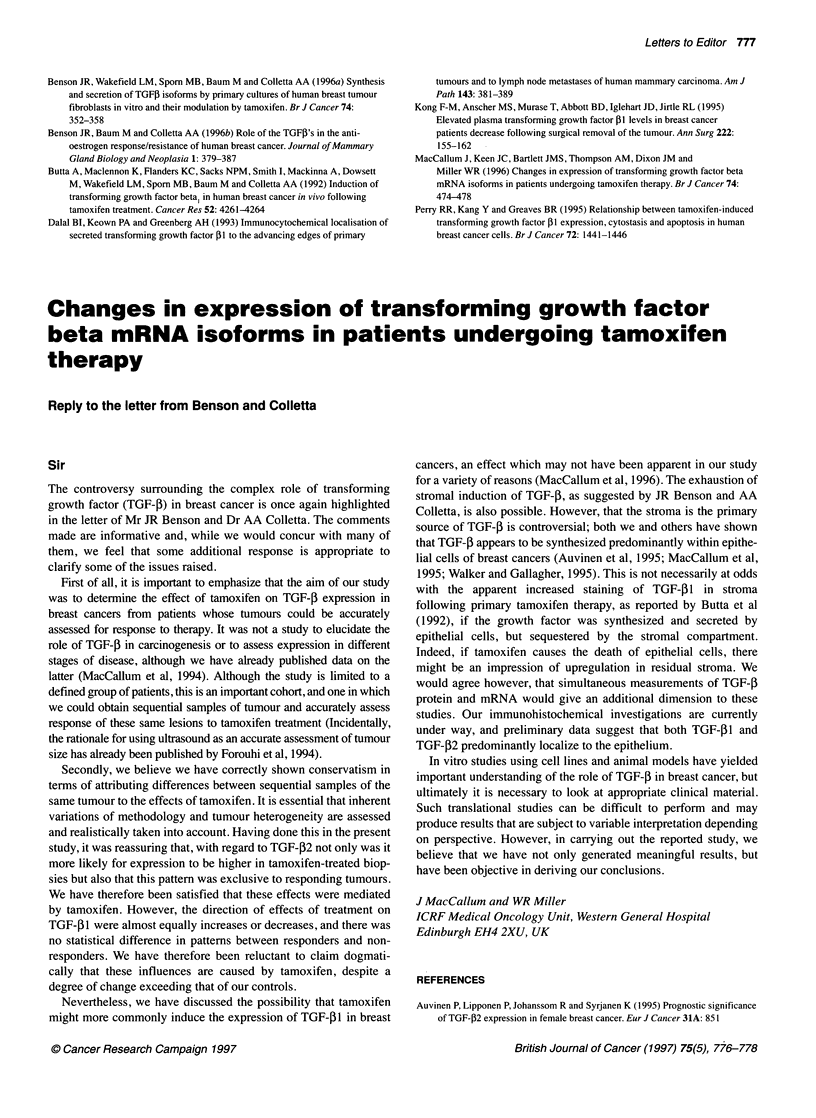

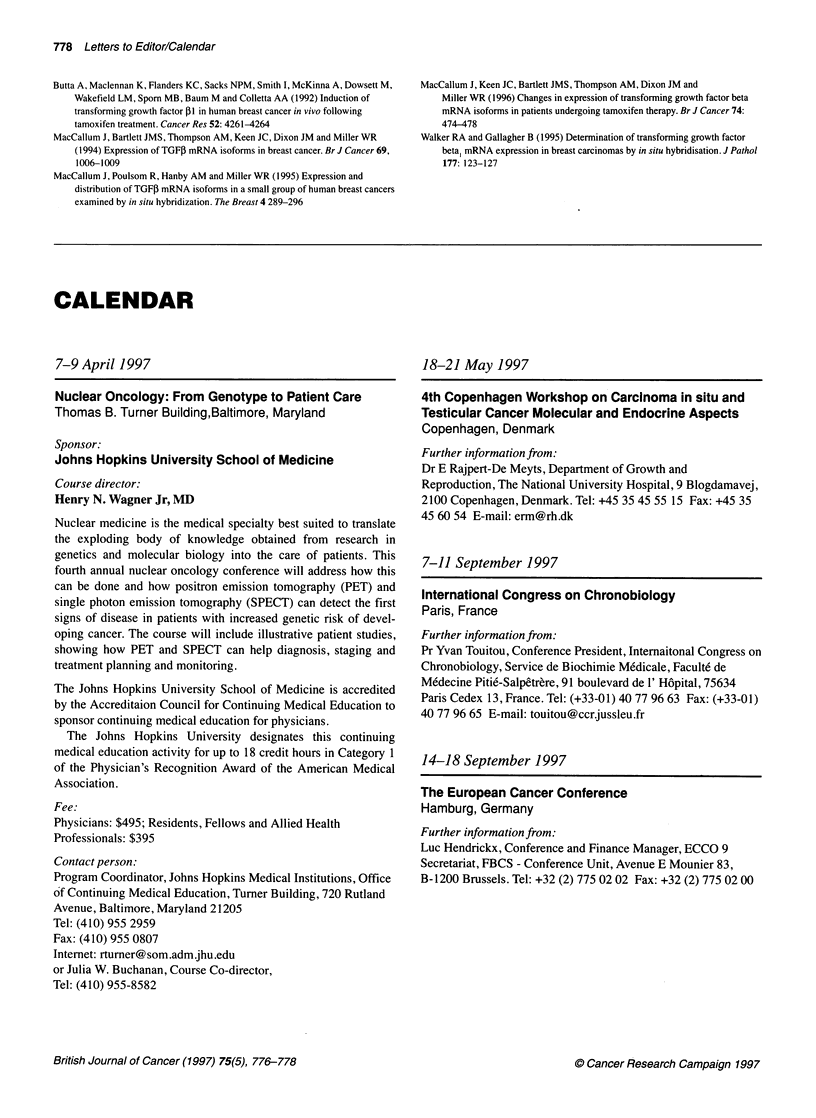


## References

[OCR_00086] Auvinen P., Lipponen P., Johansson R., Syrjänen K. (1995). Prognostic significance of TGF-beta 2 expression in female breast cancer.. Eur J Cancer.

[OCR_00094] Butta A., MacLennan K., Flanders K. C., Sacks N. P., Smith I., McKinna A., Dowsett M., Wakefield L. M., Sporn M. B., Baum M. (1992). Induction of transforming growth factor beta 1 in human breast cancer in vivo following tamoxifen treatment.. Cancer Res.

[OCR_00100] MacCallum J., Bartlett J. M., Thompson A. M., Keen J. C., Dixon J. M., Miller W. R. (1994). Expression of transforming growth factor beta mRNA isoforms in human breast cancer.. Br J Cancer.

[OCR_00110] MacCallum J., Keen J. C., Bartlett J. M., Thompson A. M., Dixon J. M., Miller W. R. (1996). Changes in expression of transforming growth factor beta mRNA isoforms in patients undergoing tamoxifen therapy.. Br J Cancer.

[OCR_00116] Walker R. A., Gallacher B. (1995). Determination of transforming growth factor beta 1 mRNA expression in breast carcinomas by in situ hybridization.. J Pathol.

